# 1,4,10,13-Tetra­oxa-7,16-diazo­nia­cyclo­octa­decane bis­(1*H*-pyrrole-2-carboxyl­ate)

**DOI:** 10.1107/S1600536813016176

**Published:** 2013-06-15

**Authors:** Fanglei Zeng, Zhenming Yin

**Affiliations:** a Tianjin Key Laboratory of Structure and Performance for Functional Molecules, Key Laboratory of Inorganic-Organic Hybrid Functional Material Chemistry, Ministry of Education, College of Chemistry, Tianjin Normal University, Tianjin 300387, People’s Republic of China

## Abstract

In the title salt, C_12_H_28_N_2_O_4_^2+^·2C_5_H_4_NO_2_^−^, the 1,4,10,13-tetra­oxa-7,16-di­aza­cyclo­octa­decane dication possesses inversion symmetry. In the crystal, the pyrrole-carboxyl­ate anions are linked *via* pairs of N—H⋯O hydrogen bonds, forming inversion dimers. These dimers are linked by the dications, *via* N—H⋯O hydrogen bonds, forming chains propagating along [110].

## Related literature

For background to hydrogen-bonded supra­molecular assemblies, see: Burrows (2004 ▶). For the hydrogen-bonded assemblies of pyrrole-based structures, see: Wang & Yin (2007 ▶); Yin & Li (2006 ▶); Cui *et al.* (2009 ▶); Li *et al.* (2012 ▶).
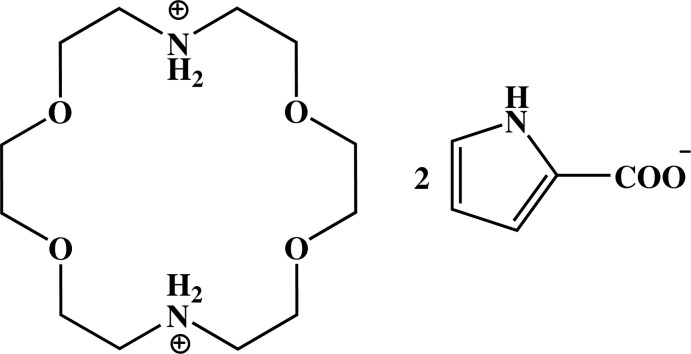


## Experimental

### 

#### Crystal data

C_12_H_28_N_2_O_4_^2+^·2C_5_H_4_NO_2_^−^
*M* *_r_* = 484.55
Triclinic, 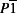

*a* = 7.8963 (19) Å
*b* = 9.164 (2) Å
*c* = 9.244 (2) Å
α = 73.028 (4)°
β = 76.547 (4)°
γ = 77.824 (4)°
*V* = 614.8 (3) Å^3^
*Z* = 1
Mo *K*α radiation
μ = 0.10 mm^−1^
*T* = 294 K
0.24 × 0.22 × 0.18 mm

#### Data collection

Bruker SMART CCD area-detector diffractometer
Absorption correction: multi-scan (*SADABS*; Bruker, 1997 ▶) *T*_min_ = 0.970, *T*_max_ = 0.982
3484 measured reflections
2471 independent reflections
1695 reflections with *I* > 2σ(*I*)
*R*_int_ = 0.017

#### Refinement

*R*[*F* ^2^ > 2σ(*F* ^2^)] = 0.042
*wR*(*F* ^2^) = 0.113
*S* = 1.03
2471 reflections
166 parameters
H atoms treated by a mixture of independent and constrained refinement
Δρ_max_ = 0.14 e Å^−3^
Δρ_min_ = −0.26 e Å^−3^

### 

Data collection: *SMART* (Bruker, 1997 ▶); cell refinement: *SAINT* (Bruker, 1997 ▶); data reduction: *SAINT*; program(s) used to solve structure: *SHELXS97* (Sheldrick, 2008 ▶); program(s) used to refine structure: *SHELXL97* (Sheldrick, 2008 ▶); molecular graphics: *SHELXTL* (Sheldrick, 2008 ▶); software used to prepare material for publication: *SHELXTL*.

## Supplementary Material

Crystal structure: contains datablock(s) global, I. DOI: 10.1107/S1600536813016176/ff2108sup1.cif

Structure factors: contains datablock(s) I. DOI: 10.1107/S1600536813016176/ff2108Isup2.hkl

Supplementary material file. DOI: 10.1107/S1600536813016176/ff2108Isup3.cml

Additional supplementary materials:  crystallographic information; 3D view; checkCIF report

## Figures and Tables

**Table 1:** Hydrogen-bond geometry (Å, °)

*D*—H⋯*A*	*D*—H	H⋯*A*	*D*⋯*A*	*D*—H⋯*A*
N2—H2⋯O4^i^	0.877 (19)	1.94 (2)	2.7741 (19)	158.0 (17)
N1—H1*B*⋯O3^ii^	0.91 (2)	2.01 (2)	2.8167 (19)	147.4 (16)
N1—H1*A*⋯O4	0.94 (2)	2.489 (19)	3.137 (2)	125.9 (14)
N1—H1*A*⋯O3	0.94 (2)	1.81 (2)	2.7452 (19)	171.1 (17)

Symmetry codes: (i) 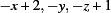; (ii) 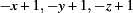.

**Fig. 1.**
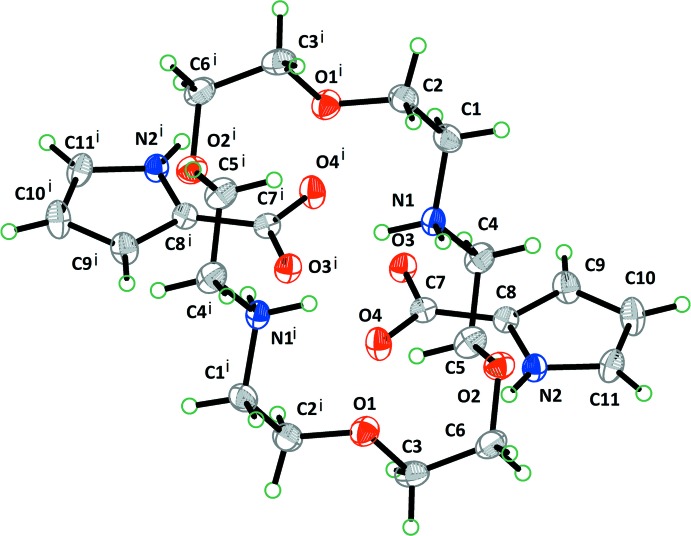
The molecular structure of (I), with the numbering scheme and 30% probability displacement ellipsoids. [Symmetry code: (i) 1-x, 1-y, 1-z]

**Fig. 2.**
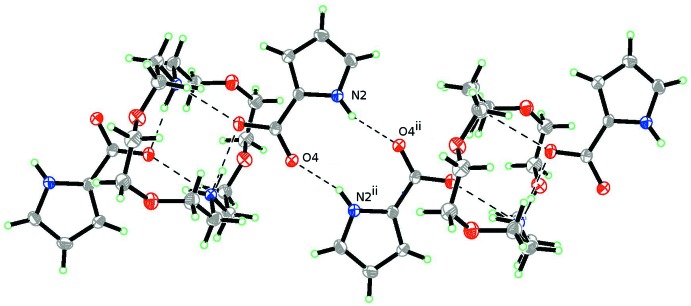
The dimer of molecules of (I) connected by N—H···O hydrogen bonds (dashed lines). [Symmetry code: (ii) -x + 2, -y, -z + 1]

## supplementary crystallographic information

### Comment

Hydrogen-bond-mediated self-assembly represents an area of considerable current interest (Burrows, 2004). It has recently been found that pyrrole-based entities are also capable of undergoing self-assembly through hydrogen bonds, especially in the solid state. In our previous works, we have reported the hydrogen-bonded assemblies of 4-pyridylmethyl 1*H*-pyrrole-2-carboxylate (Wang & Yin, 2007) and some other pyrrole-based compounds (Yin & Li, 2006; Cui *et al.* 2009; Li *et al.* 2012) in the solid state. Here we report the self-assembly of the title compound, (I), *via* conventional N—H···O hydrogen bonds.

The molecular structure of (I) is shown in Fig.1. In the solid state, the compound adopts central symmetrical conformation. Each pyrrole-2-carboxylate group is planar and interact with protonated amino group through two charge assisted N—H···O hydrogen bonds.

In the crystal structure, the molecules of (I) are held together by a pair of N—H···O hydrogen bonds between the pyrrole and carbonyl groups (Fig.2). Consequently, the molecules of (I) form a one-dimensional infinite chain structure.

### Experimental

1,4,10,13-Tetraoxa-7,16-diaza-cyclooctadecane (262 mg, 100 mmol), 1*H*-pyrrole-2-carboxylic acid (222 mg, 200 mmol) were added to alcohol (20 ml), and the mixture was stirred in room temperature for 1 h. The solution was then evaporated and afforded the title compound (colorless crystals, 387 mg, 70%).

### Refinement

The N-bound H atoms were located in a difference map and refined freely. Other H atoms were positioned geometrically (C—H = 0.93 or 0.97 A°) and refined using a riding model, with *U*_iso_(H) =1.2*U*_eq_(C).

### Crystal data

**Table:**

C_12_H_28_N_2_O_4_^2+^·2C_5_H_4_NO_2_^−^	*Z* = 1
*M**_r_* = 484.55	*F*(000) = 260
Triclinic, *P*1	*D*_x_ = 1.309 Mg m^−^^3^
Hall symbol: -P 1	Mo *K*α radiation, λ = 0.71073 Å
*a* = 7.8963 (19) Å	Cell parameters from 1288 reflections
*b* = 9.164 (2) Å	θ = 2.7–25.6°
*c* = 9.244 (2) Å	µ = 0.10 mm^−^^1^
α = 73.028 (4)°	*T* = 294 K
β = 76.547 (4)°	Block, colourless
γ = 77.824 (4)°	0.24 × 0.22 × 0.18 mm
*V* = 614.8 (3) Å^3^	

### Data collection

**Table:**

Bruker SMART CCD area-detector diffractometer	2471 independent reflections
Radiation source: fine-focus sealed tube	1695 reflections with *I* > 2σ(*I*)
Graphite monochromator	*R*_int_ = 0.017
phi and ω scans	θ_max_ = 26.3°, θ_min_ = 2.3°
Absorption correction: multi-scan (*SADABS*; Bruker, 1997)	*h* = −9→9
*T*_min_ = 0.970, *T*_max_ = 0.982	*k* = −11→7
3484 measured reflections	*l* = −11→11

### Refinement

**Table:**

Refinement on *F*^2^	Primary atom site location: structure-invariant direct methods
Least-squares matrix: full	Secondary atom site location: difference Fourier map
*R*[*F*^2^ > 2σ(*F*^2^)] = 0.042	Hydrogen site location: inferred from neighbouring sites
*wR*(*F*^2^) = 0.113	H atoms treated by a mixture of independent and constrained refinement
*S* = 1.03	*w* = 1/[σ^2^(*F*_o_^2^) + (0.0587*P*)^2^ + 0.0353*P*] where *P* = (*F*_o_^2^ + 2*F*_c_^2^)/3
2471 reflections	(Δ/σ)_max_ < 0.001
166 parameters	Δρ_max_ = 0.14 e Å^−^^3^
0 restraints	Δρ_min_ = −0.26 e Å^−^^3^

### Special details

**Table:**

Geometry. All e.s.d.'s (except the e.s.d. in the dihedral angle between two l.s. planes) are estimated using the full covariance matrix. The cell e.s.d.'s are taken into account individually in the estimation of e.s.d.'s in distances, angles and torsion angles; correlations between e.s.d.'s in cell parameters are only used when they are defined by crystal symmetry. An approximate (isotropic) treatment of cell e.s.d.'s is used for estimating e.s.d.'s involving l.s. planes.
Refinement. Refinement of *F*^2^ against ALL reflections. The weighted *R*-factor *wR* and goodness of fit *S* are based on *F*^2^, conventional *R*-factors *R* are based on *F*, with *F* set to zero for negative *F*^2^. The threshold expression of *F*^2^ > σ(*F*^2^) is used only for calculating *R*-factors(gt) *etc*. and is not relevant to the choice of reflections for refinement. *R*-factors based on *F*^2^ are statistically about twice as large as those based on *F*, and *R*- factors based on ALL data will be even larger.

### Fractional atomic coordinates and isotropic or equivalent isotropic displacement parameters (Å^2^)

**Table:**

	*x*	*y*	*z*	*U*_iso_*/*U*_eq_	
N1	0.5212 (2)	0.36442 (16)	0.71663 (16)	0.0346 (3)	
H1A	0.592 (3)	0.369 (2)	0.618 (2)	0.051 (5)*	
H1B	0.434 (3)	0.447 (2)	0.706 (2)	0.049 (5)*	
N2	0.99820 (18)	0.10017 (16)	0.27227 (16)	0.0346 (3)	
H2	1.027 (2)	0.022 (2)	0.348 (2)	0.048 (5)*	
O1	0.41888 (15)	0.19373 (14)	0.53943 (14)	0.0462 (3)	
O2	0.55409 (16)	0.64120 (13)	0.77534 (14)	0.0462 (3)	
O3	0.72871 (15)	0.40741 (12)	0.42918 (12)	0.0391 (3)	
O4	0.85227 (16)	0.17742 (13)	0.55299 (13)	0.0439 (3)	
C1	0.4410 (3)	0.2209 (2)	0.7790 (2)	0.0453 (5)	
H1C	0.3761	0.2187	0.8823	0.054*	
H1D	0.5342	0.1326	0.7857	0.054*	
C2	0.3194 (2)	0.2067 (2)	0.6832 (2)	0.0457 (5)	
H2A	0.2624	0.1163	0.7332	0.055*	
H2B	0.2290	0.2970	0.6705	0.055*	
C3	0.3199 (3)	0.1628 (2)	0.4441 (2)	0.0495 (5)	
H3A	0.2031	0.2233	0.4538	0.059*	
H3B	0.3075	0.0544	0.4754	0.059*	
C4	0.6376 (3)	0.3752 (2)	0.8169 (2)	0.0482 (5)	
H4A	0.7369	0.2923	0.8172	0.058*	
H4B	0.5729	0.3636	0.9216	0.058*	
C5	0.7031 (2)	0.5272 (2)	0.7613 (2)	0.0486 (5)	
H5A	0.7851	0.5313	0.8230	0.058*	
H5B	0.7627	0.5427	0.6548	0.058*	
C6	0.5862 (3)	0.7961 (2)	0.7188 (2)	0.0507 (5)	
H6A	0.6552	0.8153	0.7836	0.061*	
H6B	0.4742	0.8641	0.7278	0.061*	
C7	0.8236 (2)	0.27649 (18)	0.43185 (18)	0.0318 (4)	
C8	0.9026 (2)	0.24102 (18)	0.28271 (18)	0.0313 (4)	
C9	0.8985 (2)	0.3296 (2)	0.13557 (19)	0.0432 (4)	
H9	0.8426	0.4309	0.1075	0.052*	
C10	0.9934 (3)	0.2405 (2)	0.0351 (2)	0.0505 (5)	
H10	1.0120	0.2716	−0.0718	0.061*	
C11	1.0535 (2)	0.0996 (2)	0.1224 (2)	0.0437 (5)	
H11	1.1209	0.0174	0.0854	0.052*	

### Atomic displacement parameters (Å^2^)

**Table:**

	*U*^11^	*U*^22^	*U*^33^	*U*^12^	*U*^13^	*U*^23^
N1	0.0400 (8)	0.0321 (8)	0.0289 (8)	0.0031 (7)	−0.0085 (7)	−0.0080 (6)
N2	0.0376 (8)	0.0328 (8)	0.0310 (8)	0.0017 (6)	−0.0068 (6)	−0.0091 (6)
O1	0.0407 (7)	0.0546 (8)	0.0490 (8)	−0.0112 (6)	−0.0072 (6)	−0.0200 (6)
O2	0.0475 (7)	0.0390 (7)	0.0528 (8)	−0.0051 (6)	−0.0082 (6)	−0.0147 (6)
O3	0.0416 (7)	0.0323 (6)	0.0401 (7)	0.0072 (5)	−0.0095 (5)	−0.0115 (5)
O4	0.0509 (8)	0.0387 (7)	0.0324 (6)	0.0094 (6)	−0.0086 (6)	−0.0049 (5)
C1	0.0548 (12)	0.0358 (10)	0.0393 (10)	−0.0065 (8)	−0.0051 (9)	−0.0034 (8)
C2	0.0458 (11)	0.0414 (10)	0.0467 (11)	−0.0092 (8)	−0.0022 (9)	−0.0096 (8)
C3	0.0525 (12)	0.0437 (11)	0.0602 (12)	−0.0163 (9)	−0.0190 (10)	−0.0121 (9)
C4	0.0646 (13)	0.0408 (11)	0.0425 (10)	0.0004 (9)	−0.0267 (9)	−0.0086 (8)
C5	0.0481 (11)	0.0492 (11)	0.0541 (12)	−0.0011 (9)	−0.0225 (9)	−0.0157 (9)
C6	0.0632 (13)	0.0426 (11)	0.0542 (12)	−0.0095 (9)	−0.0155 (10)	−0.0196 (9)
C7	0.0281 (9)	0.0310 (9)	0.0370 (9)	−0.0019 (7)	−0.0086 (7)	−0.0092 (7)
C8	0.0283 (8)	0.0309 (8)	0.0337 (9)	−0.0002 (7)	−0.0077 (7)	−0.0082 (7)
C9	0.0443 (10)	0.0405 (10)	0.0363 (10)	0.0057 (8)	−0.0095 (8)	−0.0040 (8)
C10	0.0497 (11)	0.0651 (13)	0.0304 (9)	0.0053 (10)	−0.0089 (8)	−0.0115 (9)
C11	0.0425 (10)	0.0499 (11)	0.0404 (10)	0.0052 (8)	−0.0076 (8)	−0.0225 (9)

### Geometric parameters (Å, º)

**Table:**

N1—C1	1.483 (2)	C3—C6^i^	1.492 (3)
N1—C4	1.484 (2)	C3—H3A	0.9700
N1—H1A	0.94 (2)	C3—H3B	0.9700
N1—H1B	0.91 (2)	C4—C5	1.495 (3)
N2—C11	1.354 (2)	C4—H4A	0.9700
N2—C8	1.369 (2)	C4—H4B	0.9700
N2—H2	0.877 (19)	C5—H5A	0.9700
O1—C2	1.402 (2)	C5—H5B	0.9700
O1—C3	1.424 (2)	C6—C3^i^	1.492 (3)
O2—C5	1.408 (2)	C6—H6A	0.9700
O2—C6	1.417 (2)	C6—H6B	0.9700
O3—C7	1.2701 (18)	C7—C8	1.474 (2)
O4—C7	1.2519 (18)	C8—C9	1.369 (2)
C1—C2	1.496 (2)	C9—C10	1.397 (2)
C1—H1C	0.9700	C9—H9	0.9300
C1—H1D	0.9700	C10—C11	1.361 (3)
C2—H2A	0.9700	C10—H10	0.9300
C2—H2B	0.9700	C11—H11	0.9300
			
C1—N1—C4	111.26 (13)	C5—C4—H4A	109.5
C1—N1—H1A	112.0 (11)	N1—C4—H4B	109.5
C4—N1—H1A	106.5 (11)	C5—C4—H4B	109.5
C1—N1—H1B	109.1 (12)	H4A—C4—H4B	108.1
C4—N1—H1B	111.2 (12)	O2—C5—C4	106.59 (16)
H1A—N1—H1B	106.7 (16)	O2—C5—H5A	110.4
C11—N2—C8	109.53 (15)	C4—C5—H5A	110.4
C11—N2—H2	122.7 (12)	O2—C5—H5B	110.4
C8—N2—H2	127.7 (12)	C4—C5—H5B	110.4
C2—O1—C3	113.17 (14)	H5A—C5—H5B	108.6
C5—O2—C6	115.71 (15)	O2—C6—C3^i^	114.91 (15)
N1—C1—C2	113.14 (14)	O2—C6—H6A	108.5
N1—C1—H1C	109.0	C3^i^—C6—H6A	108.5
C2—C1—H1C	109.0	O2—C6—H6B	108.5
N1—C1—H1D	109.0	C3^i^—C6—H6B	108.5
C2—C1—H1D	109.0	H6A—C6—H6B	107.5
H1C—C1—H1D	107.8	O4—C7—O3	123.76 (15)
O1—C2—C1	108.26 (15)	O4—C7—C8	118.96 (13)
O1—C2—H2A	110.0	O3—C7—C8	117.28 (14)
C1—C2—H2A	110.0	N2—C8—C9	107.10 (14)
O1—C2—H2B	110.0	N2—C8—C7	122.19 (14)
C1—C2—H2B	110.0	C9—C8—C7	130.70 (15)
H2A—C2—H2B	108.4	C8—C9—C10	107.78 (16)
O1—C3—C6^i^	108.78 (15)	C8—C9—H9	126.1
O1—C3—H3A	109.9	C10—C9—H9	126.1
C6^i^—C3—H3A	109.9	C11—C10—C9	107.46 (15)
O1—C3—H3B	109.9	C11—C10—H10	126.3
C6^i^—C3—H3B	109.9	C9—C10—H10	126.3
H3A—C3—H3B	108.3	N2—C11—C10	108.13 (15)
N1—C4—C5	110.60 (14)	N2—C11—H11	125.9
N1—C4—H4A	109.5	C10—C11—H11	125.9
			
C4—N1—C1—C2	178.78 (15)	O4—C7—C8—N2	3.8 (2)
C3—O1—C2—C1	173.64 (14)	O3—C7—C8—N2	−175.99 (14)
N1—C1—C2—O1	64.80 (19)	O4—C7—C8—C9	−176.92 (17)
C2—O1—C3—C6^i^	160.84 (15)	O3—C7—C8—C9	3.3 (3)
C1—N1—C4—C5	−174.38 (16)	N2—C8—C9—C10	−0.1 (2)
C6—O2—C5—C4	−176.05 (14)	C7—C8—C9—C10	−179.46 (16)
N1—C4—C5—O2	63.76 (19)	C8—C9—C10—C11	0.0 (2)
C5—O2—C6—C3^i^	54.4 (2)	C8—N2—C11—C10	−0.1 (2)
C11—N2—C8—C9	0.12 (19)	C9—C10—C11—N2	0.1 (2)
C11—N2—C8—C7	179.58 (15)		

Symmetry code: (i) −*x*+1, −*y*+1, −*z*+1.

### Hydrogen-bond geometry (Å, º)

**Table:**

*D*—H···*A*	*D*—H	H···*A*	*D*···*A*	*D*—H···*A*
N2—H2···O4^ii^	0.877 (19)	1.94 (2)	2.7741 (19)	158.0 (17)
N1—H1*B*···O3^i^	0.91 (2)	2.01 (2)	2.8167 (19)	147.4 (16)
N1—H1*A*···O4	0.94 (2)	2.489 (19)	3.137 (2)	125.9 (14)
N1—H1*A*···O3	0.94 (2)	1.81 (2)	2.7452 (19)	171.1 (17)

Symmetry codes: (i) −*x*+1, −*y*+1, −*z*+1; (ii) −*x*+2, −*y*, −*z*+1.
